# New low-viscosity overlay medium for viral plaque assays

**DOI:** 10.1186/1743-422X-3-63

**Published:** 2006-08-31

**Authors:** Mikhail Matrosovich, Tatyana Matrosovich, Wolfgang Garten, Hans-Dieter Klenk

**Affiliations:** 1 Institute of Virology, Philipps University, Hans-Meerwein-Str. 3, 35043 Marburg, Germany

## Abstract

**Background:**

Plaque assays in cell culture monolayers under solid or semisolid overlay media are commonly used for quantification of viruses and antiviral substances. To overcome the pitfalls of known overlays, we tested suspensions of microcrystalline cellulose Avicel RC/CL™ as overlay media in the plaque and plaque-inhibition assay of influenza viruses.

**Results:**

Significantly larger plaques were formed under Avicel-containing media, as compared to agar and methylcellulose (MC) overlay media. The plaque size increased with decreasing Avicel concentration, but even very diluted Avicel overlays (0.3%) ensured formation of localized plaques. Due to their low viscosity, Avicel overlays were easier to use than methylcellulose overlays, especially in the 96-well culture plates. Furthermore, Avicel overlay could be applied without prior removal of the virus inoculum thus facilitating the assay and reducing chances of cross-contamination. Using neuraminidase inhibitor oseltamivir carboxylate, we demonstrated applicability of the Avicel-based plaque reduction assay for testing of antiviral substances.

**Conclusion:**

Plaque assay under Avicel-containing overlay media is easier, faster and more sensitive than assays under agar- and methylcellulose overlays. The assay can be readily performed in a 96-well plate format and seems particularly suitable for high-throughput virus titrations, serological studies and experiments on viral drug sensitivity. It may also facilitate work with highly pathogenic agents performed under hampered conditions of bio-safety labs.

## Background

Plaque assays in cell culture monolayers represent the most common method for quantification of infectious viruses and antiviral substances (see reference 1 for a review). In these assays, each infectious virus particle multiplies under conditions that result in a localized area of infected cells known as plaque. The plaques are revealed either as area of dead/destroyed cells detected by general cellular stains or as area of infected cells detected by immuno-staining.

Some viruses, such as herpes viruses and poxviruses, may be plaque-assayed under standard liquid culture medium, because direct cell-to-cell spread of these viruses ensures formation of localized plaques. However, many viruses, including the influenza virus, do not form localized plaques under liquid medium. This is because the progeny of these viruses efficiently detach from infected cells and spread over the cell monolayer with convectional flow of the liquid medium, which results from the temperature gradients in the culture vessels. In this case, large uneven foci of infected cells are formed, which cannot be counted (for examples, see Fig. 4 and Fig. 6a). To prevent liquid movement in the culture vessels and to control viral spread, special overlay media are used. The most common method is to solidify the culture medium with an agar (or agarose) gel. The solid gel overlays, however, cannot be used in 48- and 96-well culture plates required for automated high-throughput assays. As an alternative to solid gels, viscous solutions of soluble hydrophilic polymers, methylcellulose (MC), carboxymethylcellulose, tragacanth gum, etc. can be employed [1].

We recently developed a focus reduction assay of influenza virus sensitivity to neuraminidase inhibitors in 96-well format under liquid medium [2]. Our efforts to perform this assay under MC overlays were discouraging due to a high viscosity of methylcellulose-containing media. As described below, we overcame this problem by employing new overlay media based on suspensions of microcrystalline cellulose Avicel™ RC/CL (FMC Biopolymer). Avicel RC/CL is a colloidal form of water insoluble cellulose microparticles blended with sodium carboxymethylcellulose to facilitate dispersion. Microparticles of Avicel are readily dispersed in water to form suspensions and thixotropic gels used in the preparation of pharmaceutical suspensions and emulsions [3,4]. These properties of Avicel, as well as the relatively low viscosity of Avicel-containing suspensions, prompted us to test whether such suspensions can be used as a convenient overlay media in the plaque assay of the influenza virus.

## Results and discussion

### Comparison of Avicel and agar overlays

**Figure 1:**
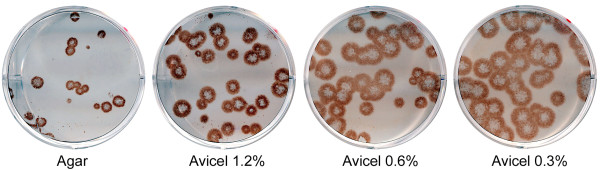
**Parallel plaque assays in 6-well plates under agar overlay and overlays containing 1.2%, 0.6%, and 0.3% of Avicel RC-581**. MDCK cells were infected with influenza virus A/Memphis/14/96-M (H1N1) and immuno-stained using aminoethylcarbazole substrate (AEC).

We first compared Avicel-based overlay media with standard agar overlay in the plaque assay in 6-well plates. Fig. 1 illustrates results that were obtained in several replicate experiments performed on different days. In all these experiments, larger plaques were formed under Avicel-containing overlays, as compared to standard agar overlay. The plaque size increased with decreasing Avicel concentration, but even diluted Avicel overlays ensured formation of well-localized plaques. Somewhat more plaques were formed under Avicel than under agar, presumably due to partial inactivation of the virus by heated agar overlay.

**Figure 2:**
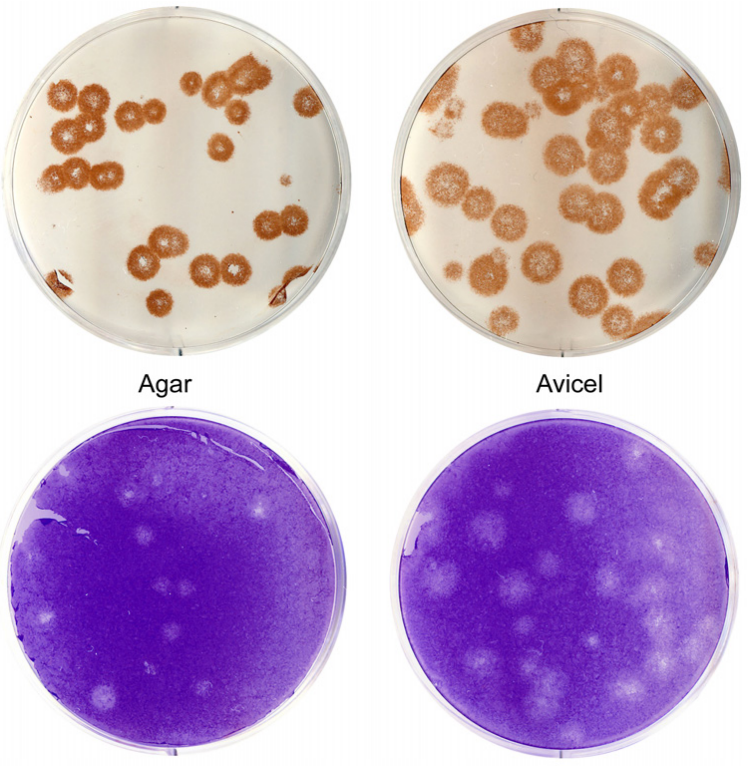
**Parallel plaque assays in 6-well plates under agar (left) and 1.2% Avicel RC-581 (right)**. Infection with A/Memphis/14/96-M (H1N1) in MDCK cells. Replicate cultures were either immuno-stained with AEC (top) or stained with crystal violet dye (bottom).

Fig. 2 shows parallel assays under agar and Avicel overlays with two commonly used methods of plaque detection, immuno-staining and staining with crystal violet, to reveal infected cells and destroyed cells, respectively. Avicel overlay performed better than agar overlay irrespective of the staining method.

**Figure 3:**
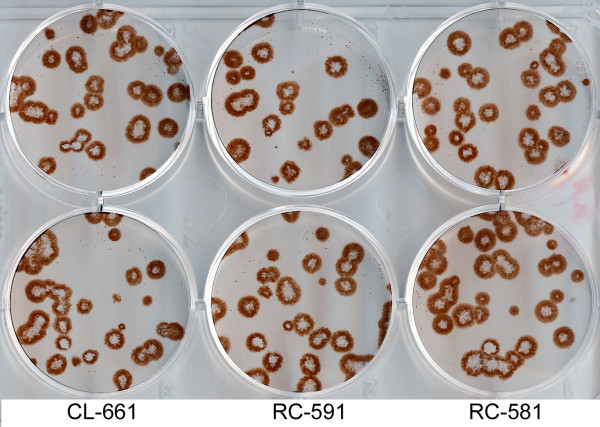
**Parallel plaque assays under overlays prepared using three different types of Avicel (CL-661, RC-591 and RC-581) with the same concentration (1.2%)**. Infection with A/Memphis/14/96-M (H1N1) in MDCK cells. Two replicate wells of the 6-well plate are shown for each overlay. Immuno-staining (AEC).

FMC BioPolymer produces three different types of hydrophylic microcrystalline cellulose, Avicel CL-661, Avicel RC-591 and Avicel RC-581 [3,4]. We compared them in the same experiment using three different concentrations for each type, 1.2% (Fig. 3), 0.6%, and 0.3% (data not shown). The plaque size was independent of the Avicel type, and the number of the plaques depended neither on the Avicel type nor concentration. We arbitrarily used Avicel RC-581 in all subsequent experiments.

### Comparison of Avicel and methylcellulose overlays

**Figure 4:**
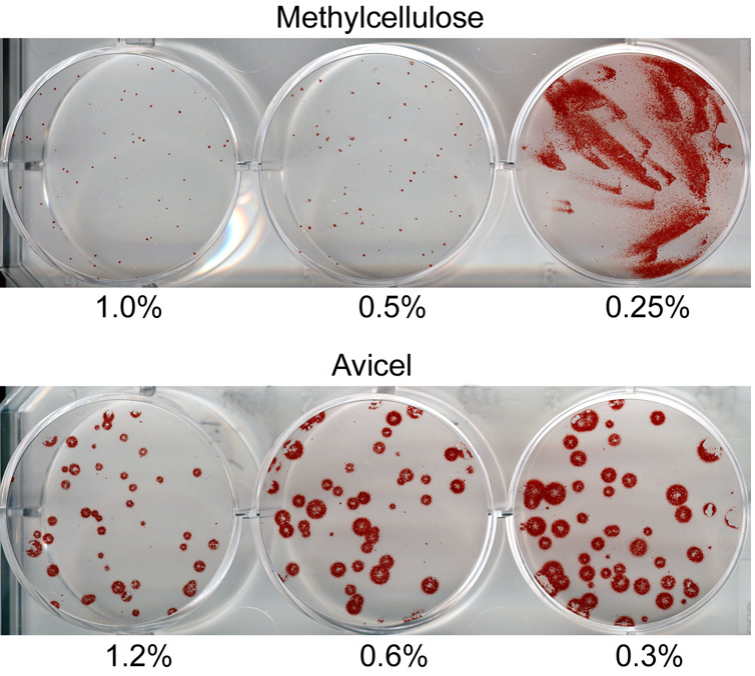
**Parallel plaque assays under methylcellulose (top panel) and Avicel RC-581 (bottom panel)**. Numbers depict concentrations of the MC and Avicel in the overlay. A/Memphis/14/96-M (H1N1); MDCK-SIAT1 cells in 6-well plates; immuno-staining (AEC).

Human influenza virus formed rather small plaques in MDCK-SIAT1 cells under 1% methylcellulose overlay (Fig. 4), in part due to inherently reduced virus spread in this genetically modified cell line [2]. Lowering MC concentration to 0.5% resulted in only marginal increase in the plaque size, whereas utilization of 0.25% MC resulted in a formation of large comet-shaped foci. This peculiar shape of foci suggested that the virus progeny is carried over the cell monolayer by convectional movement of the overlay medium [5] and that 0.25% MC overlay fails to prevent such movement. By marked contrast, even the most diluted Avicel-based overlay (0.3%) efficiently prevented convectional flows in the culture medium while still allowing relatively unrestricted radial growth of the viral plaques. As a result, plaques formed under Avicel were substantially bigger than those formed under MC. The number of plaques did not differ between the two types of overlays.

Importantly, suspensions of Avicel are much less viscous than solutions of methylcellulose used in plaque assays [1]. For example, 1.2% Avicel suspensions in water have viscosity in the range of 100–200 mPa.s [3], whereas viscosity of 2% MC solution in water is 3000–5000 mPa.s [6]. Thus, even the most concentrated Avicel overlay media tested in our experiments (1.2%) was still significantly less viscous than the least concentrated MC medium.

### Plaque formation by different influenza virus strains

**Figure 5:**
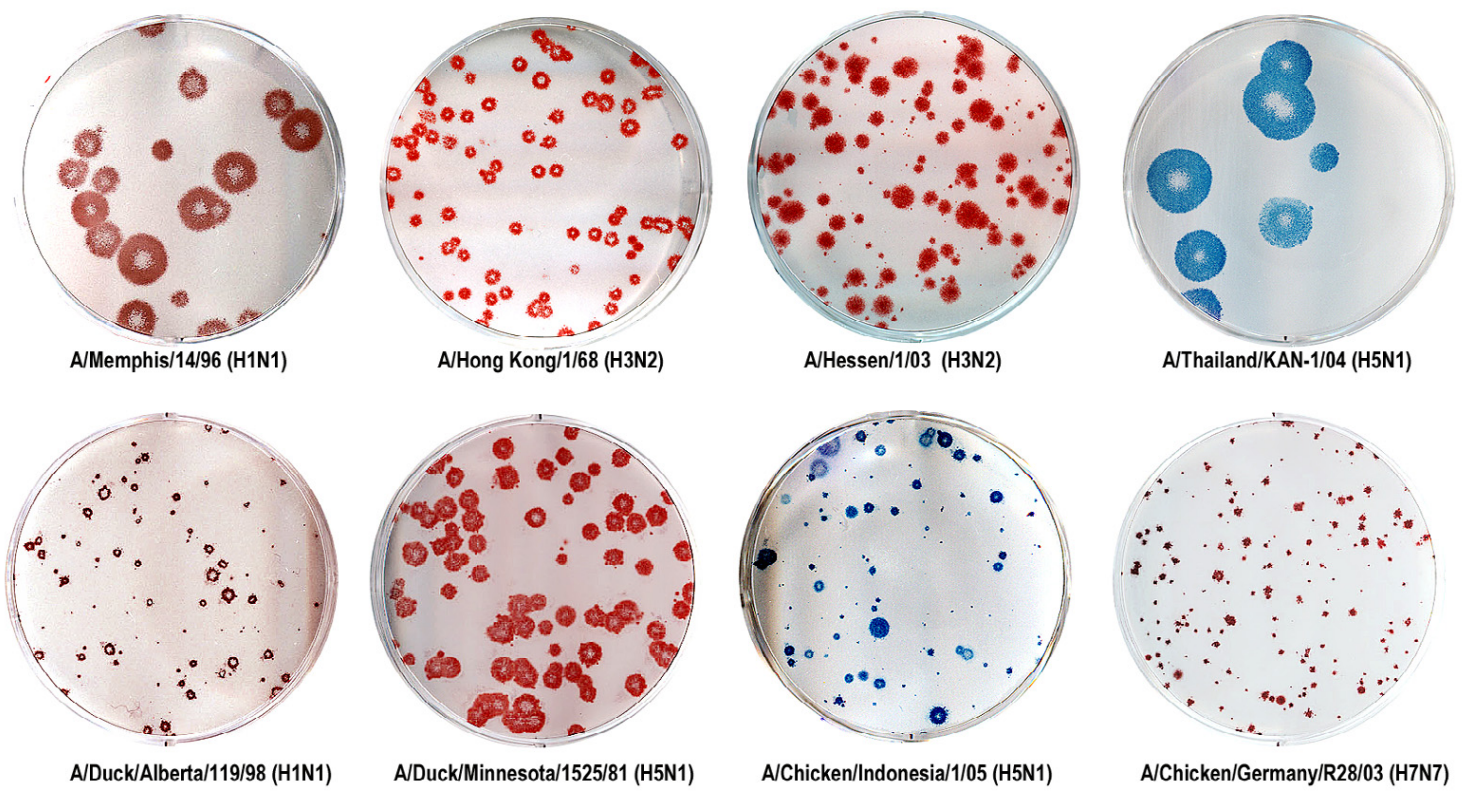
**Plaques formed by influenza viruses in MDCK cells under 1.2% Avicel RC-581 overlay**. Top row, from left to right: viruses isolated from humans A/Memphis/14/96-M (H1N1), A/Hong Kong/1/68 (H3N2), A/Hessen/1/03 (H3N2) and A/Thailand/KAN-1/04 (H5N1). Bottom row, from left to right: avian viruses A/Duck/Alberta/119/98, A/Duck/Minnesota/1525/81 (H5N1), A/Chicken/Indonesia/1/05 (H5N1) and A/Chicken/Germany/R28/03 (H7N7). Immuno-staining with either True Blue (blue) or AEC (red).

We tested eight human and avian influenza viruses belonging to four HA and three NA antigenic subtypes for the ability of these viruses to form plaques under Avicel overlay (Fig. 5). All viruses, including highly pathogenic avian H5N1 and H7N7 viruses, produced readily countable plaques. Thus, Avicel overlays appear suitable for plaque assaying of a variety of influenza virus strains irrespective of their antigenic subtype and host species.

**Figure 6:**
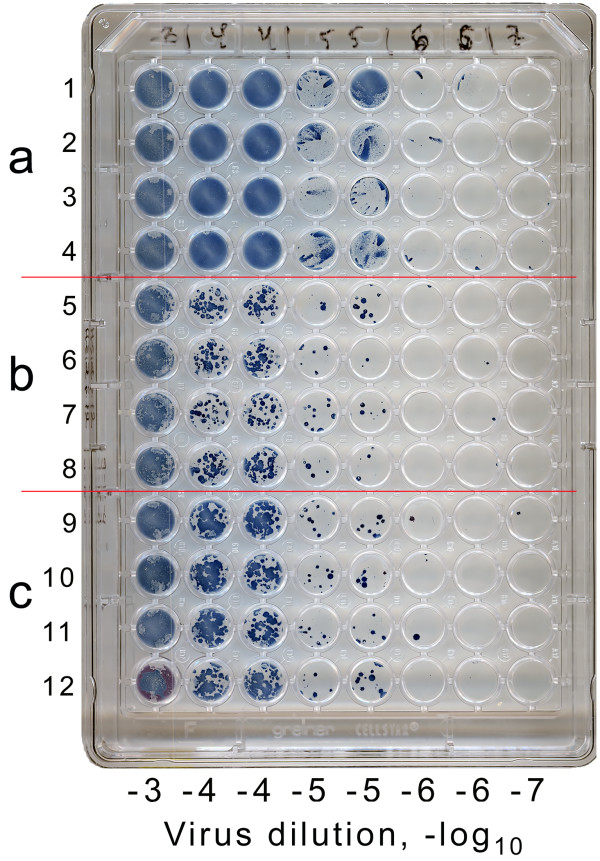
**Three different assay variants (a-c) in 96-well plate**. Either 12 or 24 replicate wells with MDCK cells were inoculated with tenfold dilutions of A/Memphis/14/96-M (H1N1) as depicted at the bottom of the figure. After 1 h of incubation, the viral inoculums were removed from the top eight rows of the wells; the inoculums were left intact in rows 9–12. After that, standard liquid maintenance medium was added to rows 1–4; 1.2% Avicel RC-581 overlay medium was added to rows 5–12. TPCK-trypsin was included in each overlay medium to produce the final concentration of 1 μg/ml. Immuno-staining 24 h post infection using True Blue substrate.

### Plaque assays under Avicel in 96-well plates

Avicel overlays were readily compatible with the 96-well microplate format. Due to their low viscosity, suspensions of Avicel could be easily dispensed to and removed from the plate wells by using the standard equipment for handling liquid culture media (pipettes, dispensers, multi-well washer manifolds, etc).

Plaque assays under agar and methylcellulose require removal of the initial viral inoculum before addition of the overlay [1]. The low viscosity of Avicel overlays allowed us to skip this procedure making the assay easier to perform and reducing chances of cross-contamination during removal of the inoculums. In the experiment illustrated in Fig. 6, we infected MDCK-SIAT1 cells in a 96-well plate with serial virus dilutions in the standard maintenance medium. One hour later, we either removed virus inoculum before adding the Avicel overlay medium (Fig. 6b, replicate rows 5–8), or added the same Avicel medium without removing the virus suspension from the wells (Fig. 6c, rows 9–12). Four replicate rows overlaid with the liquid medium served to illustrate a lack of plaque localization without the addition of Avicel (Fig. 6a). As can be seen, the simplified assay (c) was at least as sensitive as the standard assay (b), and both assays had similarly low well-to-well variation.

### Assay of virus sensitivity to antiviral drugs

**Figure 7:**
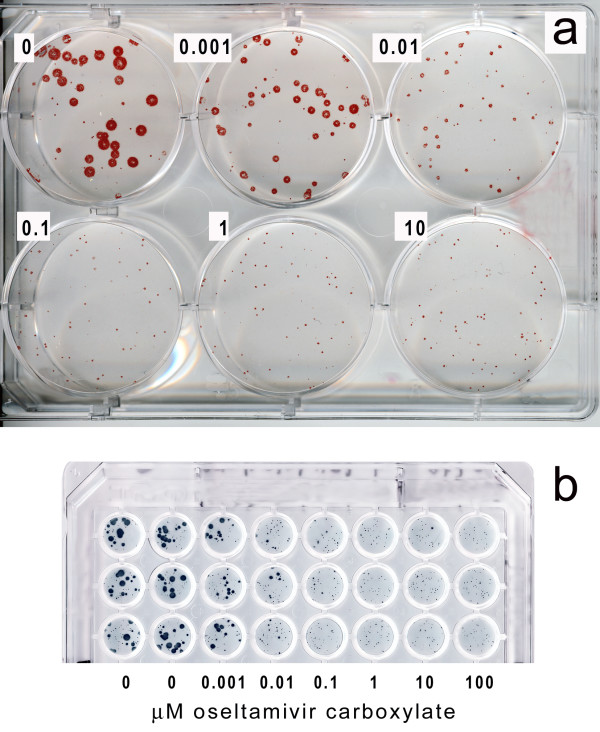
**Plaque reduction assay in MDCK-SIAT1 cells under Avicel overlay**. A/Memphis/14/96-M (H1N1) was assayed for its sensitivity to the neuraminidase inhibitor oseltamivir carboxylate in 6 well plate (**a**) and in 96-well plate (**b**; 3 replicate rows are shown in the figure) as described in the Methods section. Numbers depict final concentration of the inhibitor (μM) after the addition of the overlay.

Fig. 7 demonstrates the applicability of the simplified Avicel-based assay for testing of antiviral substances. One hour after infecting replicate MDCK-SIAT1 cultures in either 6-well or 96-well plate with the virus mixtures with variable amounts of neuraminidase inhibitor oseltamivir carboxylate, we added 1.2% Avicel overlay medium and incubated cultures for 3 days (a) and 24 hr (b), respectively. The results of the assays in 6-well plate and 96-well plate closely agreed with each other and were consistent with our previous data on sensitivity of this virus to oseltamivir carboxylate determined in the focus reduction assay [2]. In either case, the plaque size was reduced about tenfold at 0.01 μM concentration of the drug, with some reduction seen already at 0.001 μM concentration.

## Conclusion

The plaque assays under microcrystalline cellulose overlays offer several advantages over assays that utilize solid agar or semisolid MC media. Low overlay viscosity makes the assay less cumbersome, especially in a microplate format. Bigger plaque size increases sensitivity and/or reduces incubation time. Due to these advantages, Avicel-based assays appear particularly suitable for high-throughput virus titrations, serological studies and experiments on viral drug sensitivity. Although tested here with influenza virus, the assay will likely be applicable to many other viral infections, and it may substantially facilitate studies on highly pathogenic agents performed under hampered experimental conditions of bio-safety labs.

## Methods

### Virus and cells

Unless indicated otherwise, all experiments were performed using a clinical influenza virus isolate in MDCK cells A/Memphis/14/96-M (H1N1) kindly provided by Dr. Robert Webster (St. Jude Children's Hospital, USA) [2]. The viruses described in Fig. 5 were kindly provided by the colleagues listed in the Acknowledgements.

MDCK cells and MDCK-SIAT1 cells stably transfected with 2,6-sialyltransferase for enhanced expression of Neu5Ac2-6Gal-terminated oligosaccharides were described previously [2]. We propagated both types of cells in DMEM (Gibco) supplemented with 2 mM L-glutamine, 10% fetal calf serum and antibiotics (streptomycin, 100 mg/ml and penicillin, 100 U/ml).

### Overlay media

As the liquid maintenance medium (MM) for virus infection, we used Eagle's MEM containing L-glutamine, 0.1% BSA (Sigma, A-0336), antibiotics and 1 μg/ml TPCK trypsin (Sigma, T-1426).

Stocks of 1.8 % Bacto-Agar (BD, 214010) and 2% methylcellulose (Methocel MC, 3000–5500 mPa.s at 2%, Fluka) in distilled water were prepared as described previously [1].

The manufacturer of Avicel RC/CL (FMC BioPolymer) kindly provided three types of Avicel (RC-581, RC-591 and CL-661) for testing. We prepared stock suspensions of Avicel by dispersing 2.4 g Avicel powder in 100 ml distilled water on a standard magnetic stirrer for 1 hr. Agar, MC and Avicel stocks were sterilized by autoclaving for 20 min at 121°C and stored at room temperature.

The overlays were prepared by mixing stock solutions of MC, Avicel or melted agar with equal volumes of double-strength maintenance medium. To vary the concentration of MC and Avicel in the overlays, we diluted original stocks with sterile water. The agar overlay was prepared at 42–44°C, two other overlays were prepared at either 20 or 37°C.

Concentrated (2.4%) Avicel stocks typically did not separate during storage, diluted Avicel-based overlays, however, were less stable. If deemed necessary, we mixed Avicel stocks, and always mixed Avicel overlays before use by either shaking with hand or vortexing. Slow separation of overlays after their application on cell monolayers did not affect the assay results.

### Plaque assay in 6-well plates

One hour after infecting the cell monolayers with 30–50 plaque forming units of the virus in 1 ml of maintenance medium without trypsin, we removed the virus inoculum, covered the cells with 3 ml of the different overlay media and incubated cultures at 35°C in 5% CO_2 _atmosphere. In the case of MC and Avicel overlays, care was taken not to disturb the plates during the incubation period in order to avoid formation of non-even plaques. After three days of incubation, we removed the overlays and fixed the cells. Agar overlay was removed using metal spatula; MC, Avicel, and liquid overlays were removed by suction. The cells were fixed with 4% paraformaldehyde solution in MEM for 30 min at 4°C and washed with PBS. All subsequent treatments of the cells were performed at room temperature. We permeabilized the cells and simultaneously blocked residual aldehyde groups by incubating the cells for 10–20 min with 1 ml/well of solution containing 0.5 % Triton-X-100 and 20 mM glycine in PBS. We immuno-stained virus-infected cells by incubating for 1 hr with monoclonal antibodies specific for the influenza A virus nucleoprotein (kindly provided by Dr. Alexander Klimov at Centers for Disease Control, USA) followed by 1 hr incubation with peroxidase-labeled anti-mouse antibodies (DAKO, Denmark) and 30 min incubation with precipitate-forming peroxidase substrates. Solution of 10% normal horse serum and 0.05% Tween-80 in PBS was used for the preparation of working dilutions of immuno-reagents. We washed the cells after the primary and secondary antibodies by incubating them three times for 3–5 min with 0.05% Tween-80 in PBS. As peroxidase substrates, we employed either ready to use True Blue™ (KPL) or solution of aminoethylcarbazole (AEC, Sigma) (0.4 mg/ml) prepared in 0.05 M sodium acetate buffer, pH 5.5 and containing 0.03% H_2_O_2_. Stained plates were washed with tap water to stop the reaction and dried. In the case of True Blue staining, which is relatively unstable in water solutions, plates were dried inverted in order to minimize bleaching. Stained plates were scanned on a flat bed scanner and the data were acquired by Adobe Photoshop 7.0 software.

As an alternative to immuno-staining, in some experiments we revealed plaques as areas of destroyed cells. To this end, after removing the overlays, we stained the cells with 1% crystal violet solution in 20% methanol in water.

### Variants of virus titration in 96-well plates

#### 1. Standard variant

We infected MDCK or MDCK-SIAT1 cell monolayers in 96-well plates with 50 μl/well of serial dilutions of the virus in the maintenance medium without trypsin. After 1 h incubation at 35°C in 5% CO2, we removed the virus, added 100 μl/well of either MC or Avicel overlay media, and incubated the cells for further 24 h to allow plaque formation. Fixation and immuno-staining were performed as described above for 6-well plates but using 50 μl of reagents per well.

#### 2. Simplified variant

The assay was performed exactly as in the standard variant with the following modifications. After 1 h incubation with the virus, we did not remove the inoculum. We added 100 μl/well of Avicel overlay media and mixed the plate either by tapping with hand or using a plate mixer. The media included increased amounts of trypsin (1.5 μg/ml) to compensate for the dilution of the overlay with the virus-containing material present in the wells.

### Plaque inhibition assay

Noel Roberts (Roche Products, UK) kindly provided the neuraminidase inhibitor oseltamivir carboxylate (OC). We added 50 μl/well of serial 10-fold dilutions of OC in maintenance medium without trypsin (concentration range from 4 nM to 400 μM OC) to monolayers of MDCK-SIAT1 cells in 96-well plates. We then added 50 μl/well of virus dilutions (20–40 plaque forming units per well). We mixed the plate and incubated it for 1 h at 35°C to allow the initiation of viral infection. After that, we added 100 μl/well of 1.2% Avicel overlay media containing 2 μg/ml trypsin, incubated cultures for 24 h and fixed and immuno-stained them as described above.

In the case of 6-well plates, we added 0.75 ml aliquots of the drug and the inhibitor and 1.5 ml aliquot of Avicel per well and immuno-stained the plaques 3 days after infection.

## Abbreviations

AEC, aminoethylcarbazole; MC, methylcellulose; MM, maintenance medium; OC, oseltamivir carboxylate.

## Competing interests

The authors declare that they have no competing interests.

## Authors' contributions

Conception and design of the study, initial experiments, manuscript preparation (MM); experimental work (TM); co-ordination and financial support of the study, manuscript preparation (WG and HDK).
